# Electroacupuncture improves hypoxic stress and energy metabolism to alleviate vascular cognitive impairment through activation of the HIF-1α/p53/NGB signaling pathway in rats

**DOI:** 10.22038/ijbms.2025.86988.18796

**Published:** 2026

**Authors:** Peijia Hu, Fangyuan Xu, Wendong Zhang, Lin Bai, Fan Dai, Yu Ye, Jingji Wang, Hongliang Cheng

**Affiliations:** 1 The Second Affiliated Hospital of Anhui University of Chinese Medicine, Hefei, China; 2 The First Clinical Medical School, Anhui University of Chinese Medicine, Hefei, China

**Keywords:** Electroacupuncture, Energy metabolism, HIF-1α, Hypoxic stress, Vascular cognitive - impairment

## Abstract

**Objective(s):**

We aimed to demonstrate that electroacupuncture (EA) alleviates vascular cognitive impairment (VCI) induced by cerebral ischemia in rats by modulating oxygen homeostasis and energy metabolism through the HIF-1α/p53/NGB signaling pathway.

**Materials and Methods:**

Male Sprague‒Dawley rats underwent bilateral common carotid artery occlusion (BCCAO) to establish a VCI model. EA was administered once daily for 30 min over two weeks. Thirty minutes prior to EA, the hypoxia-inducible factor-1α (HIF-1α) inhibitor 2-methoxyestradiol (2ME2) was injected intraperitoneally. Cognitive function following BCCAO and EA was assessed using the Morris water maze test. Western blotting was performed to analyze the protein expression of HIF-1α, heme oxygenase-1 (HO-1), and neuroglobin (NGB). In addition, p53 mRNA expression was quantified by real-time PCR, and energy metabolite levels were determined using ELISA.

**Results:**

EA significantly improved learning and memory in VCI model rats. Histopathological analysis revealed that EA attenuated neuronal apoptosis and ultrastructural damage in the cortex and hippocampus. EA upregulated HIF-1α, NGB, and HO-1 expression but downregulated p53 mRNA expression in these regions. Moreover, EA treatment reversed the expression of glucose, lactic acid, and acetone aldehyde. Notably, the beneficial effects of EA on cerebral energy metabolism were abolished by 2ME2 in VCI model rats.

**Conclusion:**

EA alleviated BCCAO-induced neurological impairment and cognitive dysfunction in rats, possibly by reducing hypoxic stress and enhancing energy metabolism in the cortex and hippocampus, potentially through modulation of the HIF-1α/p53/NGB signaling pathway.

## Introduction

Vascular cognitive impairment (VCI) is a cognitive dysfunction syndrome resulting from cerebral ischemia and hypoxic injury caused by cerebrovascular disease. Its incidence increases with age, making it a major global public health challenge (1). VCI is the second most common cause of dementia after Alzheimer’s disease (2). Therefore, identifying effective strategies for the treatment and prevention of VCI is highly important.

Chronic cerebral hypoperfusion (CCH) is widely recognized as the primary etiology of VCI. Its underlying pathological mechanism involves cerebral ischemia and hypoxia, which impair energy metabolism in critical brain regions, particularly the hippocampus and cortex, leading to neuronal damage and cognitive dysfunction (3, 4). Hypoxia-inducible factor-1α (HIF-1α), a key regulator of neuronal oxygen homeostasis and energy metabolism, plays a pivotal role in this process (5). HIF-1α-associated signaling pathways and downstream targets, including vascular endothelial growth factor (VEGF) and p53, have been shown to exert neuroprotective effects against cerebral ischemic injury (6, 7). In addition, HIF-1α upregulates neuroglobin (NGB) expression in the telencephalon, facilitating oxygen transport and providing protection against neurological disorders, including Alzheimer’s disease, Huntington’s disease, cerebral ischemia, and hypoxia (5, 8). Moreover, HIF-1α regulates cerebral energy metabolism and increases cellular viability under hypoxic conditions. Taken together, HIF-1α-mediated modulation of hypoxic stress and energy metabolism represents a crucial mechanism underlying functional recovery in VCI models.

Acupuncture, a traditional Chinese medicine therapy, has shown considerable efficacy in improving cognitive function in patients (9, 10). Preclinical studies using animal models have consistently demonstrated that electroacupuncture (EA) can improve learning and memory in rats with cognitive impairment (11-14). Despite these promising findings, the mechanisms underlying the therapeutic effects of EA remain incompletely understood. In particular, the potential involvement of HIF-1α-associated signaling pathways in mediating the beneficial effects of EA on VCI in a rat model warrants further investigation.

Previous studies have demonstrated that HIF-1α and its associated signaling pathways play critical roles in promoting cellular survival by regulating glucose metabolism and maintaining reactive oxygen species (ROS) homeostasis (6, 15). In particular, the activation of HIF-1α-mediated pathways has been shown to protect hippocampal neurons from hypoxia-induced apoptosis (16). Building on these findings, this study aimed to investigate the therapeutic mechanisms of EA in VCI model rats, with a specific focus on the HIF-1α/p53/NGB signaling pathway and its potential roles in alleviating hypoxic stress, regulating energy metabolism, reducing neuronal damage, and improving cognitive function.

## Materials and Methods

### Animals

In this study, 56 male Sprague‒Dawley rats weighing 270–300 g were obtained from Jinan Pengyue Experimental Animal Breeding Co., Ltd. (animal license number: SCXK 2014-0007). The rats were housed at 22±2 °C and 50±10% humidity under a 12-hr light/dark cycle. All experimental procedures were conducted in accordance with the Guidelines for the Care and Use of Laboratory Animals issued by the Ministry of Science and Technology. The study was approved by the Ethics Committee of Anhui University of Chinese Medicine (Ethics number: AHUCM-rats-2023067).

### Bilateral common carotid artery occlusion and experimental design

Bilateral common carotid artery occlusion (BCCAO) is a model widely used in the study of VCI. The rats were anesthetized with pentobarbital sodium (40 mg/kg, IP) and placed in a supine position. Afterward, the bilateral common carotid arteries were carefully exposed and permanently ligated (17). Sham-operated rats underwent the same procedure without arterial occlusion. Following surgery, rats exhibiting cognitive impairment were screened using the Morris water maze test. In Experiment 1, to evaluate the VCI model and the effects of EA, the rats were randomly assigned to a sham operation group (Sham), a model group (Model), or an EA treatment group (EA), with eight animals in each group. In Experiment 2, to investigate the effect of the HIF-1α inhibitor on EA efficacy, eight animals were included in each of the following groups: sham, model, EA, and 2ME2 (HIF-1α inhibitor) + EA.

### EA and drug injection

EA treatment commenced on the fourth day after surgery. The rats were restrained on a custom-made board, with their heads fixed to keep them awake. Acupoints Baihui (GV 20) and Fengfu (GV 16) were selected based on human acupoint locations and adapted to the skeletal and body structure of the rats. Needles (φ0.3 mm × 15 mm) were inserted obliquely to a depth of 5 mm and connected to an electronic acupuncture apparatus (Huatuo SDZ-V) for stimulation. EA was administered once daily for 30 min over two weeks. Following the treatment period, the rats were euthanized.

The HIF-1α inhibitor 2-methoxyestradiol (2ME2; Sigma‒Aldrich, St. Louis, MO, USA) was dissolved in PBS containing 10% dimethyl sulfoxide (DMSO). Rats received an intraperitoneal injection of 2ME2 (15 mg/kg) 30 min prior to EA treatment (18).

### Behavioral tests

All rats underwent the Morris water maze test after BCCAO and EA treatments. The maze consisted of a circular tank (diameter: 160 cm) divided into four quadrants, with a platform (diameter: 11 cm; height: 29 cm) placed at the center of one quadrant. Each rat received three training trials per day for five consecutive days, with a maximum of 120 sec to locate the hidden platform. Upon finding the platform, the rat was allowed to remain on it for 30 sec, and the escape latency was recorded. On the sixth day, the platform was removed for the probe trial, during which time the rats were allowed to swim freely for 120 sec. The time spent in the target quadrant was recorded to assess spatial memory performance.

### TUNEL staining and apoptosis measurement

After the rats were euthanized, the hippocampus and cortex of the right hemisphere from four rats were removed and fixed in 4% formaldehyde, followed by cryoprotection and coronal sectioning at 20 μm thickness. Paraffinized sections were routinely dewaxed, rehydrated, digested with trypsin for 40 min, and then washed three times with PBS. TUNEL staining was performed according to the manufacturer’s instructions, followed by mounting and coverslipping. After DAB staining, the apoptotic cells appeared brown under the microscope. The neuronal apoptosis rate was calculated as follows: neuronal apoptotic cells/total number of neuronal cells×100%.

### Transmission electron microscopy

Following euthanasia, the hippocampus and cortex of the right hemisphere of four rats were removed and fixed in 2.5% glutaraldehyde, embedded in blocks, and sectioned into ultrathin slices (~70 nm). The ultrastructures of the cortex and hippocampus were examined by transmission electron microscopy (Hitachi 7700) after lead and uranium staining.

### Western blotting

Following euthanasia, the left hippocampus and cortex of eight rats were removed and stored at -80 °C for Western blotting, real-time PCR and ELISA*. *Proteins were extracted from the left hippocampus and cortex of four rat brains using lysis buffer. Protein concentrations were determined with a BCA assay. Samples (50 μg) were separated by SDS‒PAGE, transferred onto PVDF membranes, blocked, and incubated with the following primary antibodies: anti-HIF-1α (1:300; Bioss), anti-HO-1 (1:1000; Abcam), anti-NGB (1:500; Proteintech), and anti-β-actin (1:1000; Zs-BIO). The membranes were then incubated with goat anti-rabbit IgG secondary antibodies. The protein bands were visualized using ECL chemiluminescence and quantified with ImageJ software (NIH).

### Real-time PCR

Total RNA extracted from the left hippocampus and cortex of four rat brains was reverse transcribed into cDNA (reverse transcription kit, # K1622; Thermo Scientific). The reverse transcription conditions were 42 °C for 60 min and 70 °C for 5 min, and the PCR amplification conditions were 95 °C for 1 min followed by 40 cycles of 95 °C for 20 sec and 60 °C for 1 min. The primer sequences used in this study were as follows: β-actin, forward primer (sequence 5’−3’) (AGTGTGACGTTGACATCCGT); reverse primer (sequence 5’−3’) (CAAGTCCAGACGCATGATGG); p53, forward primer (sequence 5’−3’) (CCTCTGGACGTACAACTGGT); and reverse primer (sequence 5’−3’) (CAAGTCCAGACGCATGATGG). The relative expression of p53 mRNA was calculated by the 2^-△△^^Ct^ method, with β-actin used as the internal reference.

### Enzyme-linked immunosorbent assays

Appropriate homogenates and supernatants from the left hippocampus and cortex of six rats were taken for the measurement of glucose, lactic acid and pyruvate levels. These parameters were measured using ELISA kits (product numbers F006, A019-2, and A081, respectively). Sample concentrations were calculated based on the standard curves generated from known standard concentrations and their corresponding optical density (OD) values. The signals were detected using an ELISA reader at a wavelength of 505 nm for glucose, pyruvic acid and lactic acid.

### Statistical analysis

All data are presented as means ± SDs. Statistical analyses were performed using GraphPad Prism 8.2. Escape latency was analyzed by two-way repeated-measures ANOVA with Sidak’s *post hoc* correction. The apoptosis rates and protein expression data were analyzed using one-way ANOVA followed by Bonferroni’s *post hoc* correction. A *P*-value<0.05 was considered to indicate statistical significance.

## Results

### EA improved learning, cognitive function, and neuronal integrity in the cortex and hippocampus of VCI model rats

To investigate the effects of EA on learning and cognitive function, VCI model rats underwent a 2-week EA treatment followed by the Morris water maze test. Four days after BCCAO, rats in the model group exhibited significantly longer escape latencies and shorter residence times in the target quadrant compared to those in the sham group, confirming the successful induction of VCI. After 2 weeks, the model group continued to show impaired performance relative to that of the sham control group. In contrast, compared with the model group, the EA-treated group demonstrated significantly reduced escape latencies and increased target quadrant residence times (Figure 2A–B, D). The results also revealed no significant difference in travel speed between the groups (Figure 2C).

TUNEL staining and electron microscopy revealed increased neuronal apoptosis and pronounced mitochondrial damage in the cortex and hippocampus of model rats compared with those in the sham control group; however, these pathological changes were markedly ameliorated in the EA group (Figure 2E–H). These findings indicate that BCCAO induces cognitive deficits and neuronal injury in rats, whereas EA mitigates neuronal damage and improves learning and memory.

### EA ameliorated hypoxic stress and energy metabolism disturbances in the cortex and hippocampus of VCI model rats

We assessed the effects of EA on hypoxia-related proteins and energy metabolism in the cortex and hippocampus of VCI model rats. After BCCAO, HIF-1α and HO-1 expression was significantly elevated in both regions compared with that in the sham controls (Figure 3A–F). In addition, glucose levels were reduced, whereas lactate and pyruvate levels were increased in the cortex and hippocampus (Figure 3G–L). EA treatment further increased HIF-1α and HO-1 expression, as well as glucose concentrations (Figure 3A-G, J), but significantly decreased lactate and pyruvate levels (Figure 3H, I, K, L). Correlation analysis revealed a positive relationship between HIF-1α, HO-1, and glucose expression (Figure S1). Collectively, these results indicate that EA effectively mitigated hypoxic stress and restored energy metabolism in the cortex and hippocampus of VCI model rats.

### HIF-1α inhibition reversed the protective effects of EA on learning, cognitive function, and neuronal integrity in the cortex and hippocampus of VCI model rats

To investigate the role of HIF-1α in EA-mediated improvements in cognitive function and neuronal injury, VCI model rats were treated with the HIF-1α inhibitor 2ME2 in combination with EA. As shown in Figure 4A-D, compared with the sham group, the model group exhibited an extended escape latency and a reduced time spent in the target quadrant; however, the travel speed did not differ significantly between the groups. EA significantly reduced escape latency and increased target quadrant dwell time, whereas 2ME2 administration markedly reversed these improvements. TUNEL staining and electron microscopy revealed that EA decreased cortical and hippocampal neuronal apoptosis (Figure 4E-G) and mitigated mitochondrial damage (Figure 4H); these protective effects were abolished by 2ME2 (Figure 4E-H). These findings indicate that HIF-1α is essential for the neuroprotective and cognitive benefits of EA in VCI model rats.

### HIF-1α inhibition reversed the effects of EA on hypoxic stress and energy metabolism in the cortex and hippocampus of VCI model rats

To further examine the role of HIF-1α in mediating the effects of EA, we assessed HO-1 expression and the levels of glucose, lactate, and pyruvate in the cortex and hippocampus. As shown in Figure 5, EA significantly increased HO-1 expression (Figure 5A-C) and glucose concentrations (Figure 5D, G) but reduced lactate and pyruvate levels (Figure 5E-H, F-I) in ischemic brain regions. These beneficial effects were markedly attenuated by the administration of the HIF-1α inhibitor 2ME2 (Figure 5A–I). These findings indicate that HIF-1α is critical for the EA-mediated amelioration of hypoxic stress and the restoration of energy metabolism in the cortex and hippocampus of VCI model rats.

### HIF-1α inhibition abolished the effects of EA on HIF-1α/p53/NGB signaling in the cortex and hippocampus of VCI model rats

To investigate whether EA exerts protective effects through HIF-1α-related signaling, we measured HIF-1α and NGB protein levels, as well as p53 mRNA expression, in the cortex and hippocampus of the rats. Compared with those in sham control rats, HIF-1α, NGB, and p53 levels in VCI model rats were significantly elevated. EA treatment further increased HIF-1α and NGB expression while reducing p53 mRNA levels in both regions (Figure 6A-H). Importantly, these EA-induced effects were completely abolished by the HIF-1α inhibitor 2ME2, indicating that the EA’s ability to modulate the HIF-1α/p53/NGB pathway is dependent on HIF-1α activity.

## Discussion

This study demonstrated the neuroprotective effects of EA in the BCCAO-induced VCI model rats. Although BCCAO can lead to cerebral hypoperfusion, numerous authoritative studies using this model have demonstrated that BCCAO primarily induces cognitive dysfunction resulting from damage to the forebrain and hippocampus, without causing significant motor defects (19, 20). In this experiment, the average swimming speed of the BCCAO group was slightly lower than that of the sham group, but this difference was not statistically significant. We acknowledge that this may be a nonspecific manifestation of CCH. These findings directly demonstrate that dyskinesia is not the primary contributor to the water maze behavioral phenotype in this study; rather, it reflects the spatial learning and memory impairment induced by BCCAO. EA ameliorated spatial learning and memory deficits and attenuated damage to cortical and hippocampal structures. This finding is essentially similar to the results of Yang *et al*. (20). The therapeutic effects of EA may involve the modulation of HIF-1α/p53/NGB signaling, leading to improved hypoxic stress and energy metabolism in brain tissue. EA increased the expression of HIF-1α, NGB, and HO-1 in the cortex and hippocampus but reduced p53 levels. Moreover, EA increased the levels of glucose, lactate, and pyruvate. Additionally, the positive effects of EA on learning, cognitive function, neuronal integrity, hypoxic stress, and energy metabolism were reversed by the HIF-1α inhibitor 2ME2.

VCI, characterized by cognitive deficits, is a neurodegenerative disorder caused by chronic reductions in cerebral blood flow resulting from cerebrovascular disease. Acupuncture, a traditional Chinese medicine therapy, has been employed to alleviate cognitive impairment (9, 10). In traditional Chinese medicine, VCI is associated with the Du meridian. Notably, Baihui (GV 20) and Fengfu (GV 16) are Du meridian acupoints located in the cerebral region, commonly used to treat brain-related disorders. EA stimulation of these acupoints can affect Tongdu Tiaoshen and Jiannao Shengsui, thereby improving cognitive function in patients with VCI. Our findings demonstrate that EA at these acupoints significantly ameliorates cognitive deficits in VCI model rats. Recent studies have consistently demonstrated that stimulation of GV 20 and related acupoints enhances the activation of cognition-related neurons in the cerebral cortex and mitigates cognitive impairment caused by cerebral ischemia (21, 22).

The hippocampus is a critical brain structure involved in learning and memory, and it regulates memory storage through synaptic interactions. Cognitive deficits are closely linked to neuronal damage in the cortex and hippocampus caused by ischemia and hypoxia (3, 4), whereas inhibition of neuronal apoptosis in these regions can attenuate VCI (23, 24). In this study, we observed increased apoptosis and ultrastructural damage in the cortex and hippocampus of VCI model rats, which is consistent with the findings of Dai *et al*. (11). Notably, the neuroprotective effects of EA in VCI model rats were abolished by the HIF-1α inhibitor 2ME2.

Although neuroinflammation, apoptosis, and blood–brain barrier disruption are recognized pathological mechanisms of VCI, recent studies on cerebral ischemic injury suggest that hypoxic stress and impaired brain energy metabolism also play critical roles in cognitive deficits (25). Increasing evidence indicates that HIF-1α mediates endogenous adaptive responses by modulating multiple signaling pathways under hypoxic conditions (5, 6, 14). HIF-1α-associated target genes and signaling pathways in neurons have been shown to confer neuroprotection following cerebral ischemia. For instance, Zhao *et al*. (26) reported elevated HIF-1α expression in cecal ligation and puncture (CLP)-induced hippocampus-dependent cognitive deficits. Upon activation, HIF-1α participates in the hypoxic response, promoting the transcription of downstream genes involved in adaptation and survival, such as VEGF and p53 (16, 27). Conversely, inhibition of HIF-1α accelerates apoptosis under hypoxic stress (28). P53, a tumor suppressor protein, interacts with HIF-1α to facilitate the elimination of stressed cells and help histiocytes adapt to hypoxic conditions (7). However, p53 also transcriptionally activates multiple proapoptotic BCL-2 family genes, leading to mitochondrial apoptosis. Therefore, the upregulation of HIF-1α expression combined with the downregulation of p53 expression may represent a key mechanism for reducing neuronal ischemic injury.

HIF-1α plays a pivotal role in regulating NGB expression in the telencephalon (8). Neuroglobin (NGB), an oxygen-binding heme protein primarily expressed in the brain, is involved in the neuronal response to hypoxic stress (29). NGB not only facilitates oxygen uptake and transport in neurons but also mitigates neuronal damage caused by ischemia and hypoxia (30, 31). Similarly, HO-1 expression is elevated following cerebral ischemia, where it inhibits oxidative stress and neuronal apoptosis, thereby ameliorating ischemic injury and drug-induced cognitive deficits (32,33). Activation of HIF-1α has also been shown to induce HO-1 expression and confer protection in other tissues, such as lung tissue (34). Thus, upregulating NGB and HO-1 in the brain represents a promising strategy to protect neurons from ischemic injury. In our study, EA significantly increased the expression of HIF-1α, NGB, and HO-1, while reducing p53 mRNA levels, resulting in improvements in learning and memory in VCI model rats. Notably, these beneficial effects were attenuated by the HIF-1α inhibitor 2ME2. These findings suggest that EA may ameliorate cognitive impairment by enhancing hypoxic adaptation and energy metabolism in brain tissue through the HIF-1α/p53/NGB signaling pathway, which is consistent with the findings of previous reports (16).

Furthermore, HIF-1α plays a critical role in regulating neuronal energy metabolism (35). Neuronal function, viability, and structural and functional integrity rely heavily on a continuous energy supply, which in turn depends on adequate glucose metabolism. During cerebral ischemia, insufficient glucose availability leads to neuronal necrosis in the ischemic core, highlighting the essential role of glucose metabolism following stroke (36). Following ischemic injury, the activation of HIF-1α promotes neuronal survival by enhancing glucose transport and glycolysis, maintaining redox balance, and increasing glucose uptake (37). Consistent with these findings, our study demonstrated a strong correlation between HIF-1α expression and glucose levels in the rat cortex and hippocampus (Figure S1).

Although lactate can modulate microglial inflammatory responses and alleviate ischemic brain injury (38), impaired gluconeogenesis results in lactic acidosis and excessive ROS production, ultimately inducing apoptosis (39). The accumulation of toxic metabolites further exacerbates neuronal dysfunction and CNS injury. In cerebral ischemic injury model animals, the levels of glycolytic intermediates such as lactate and pyruvate decrease despite elevated glucose levels (40). Thus, reducing toxic metabolite accumulation in brain tissue may mitigate apoptosis, improve cognitive impairment, and exert neuroprotective effects. In this study, we revealed that EA increased glucose content while reducing lactate and pyruvate levels in the cortex and hippocampus of VCI model rats. Importantly, these effects were abolished by the HIF-1α inhibitor 2ME2, suggesting that EA improves brain energy metabolism by enhancing glucose transport as well as lactate uptake and utilization, likely through the upregulation of HIF-1α and its associated signaling pathways.

Current research is limited by the inability to directly detect key metabolites such as choline, monoamines, and amino acids associated with cerebral ischemic injury. Moreover, vascular changes and immunofluorescence analyses that assess the colocalization of HIF-1α with related proteins have not been conducted. To better elucidate the therapeutic potential of acupuncture in VCI, future studies should comprehensively investigate its effects on the pathology of cerebral ischemic injury and clarify the mechanisms through which acupuncture activates HIF-1α in the brain tissue of VCI model rats.

**Figure 1:**
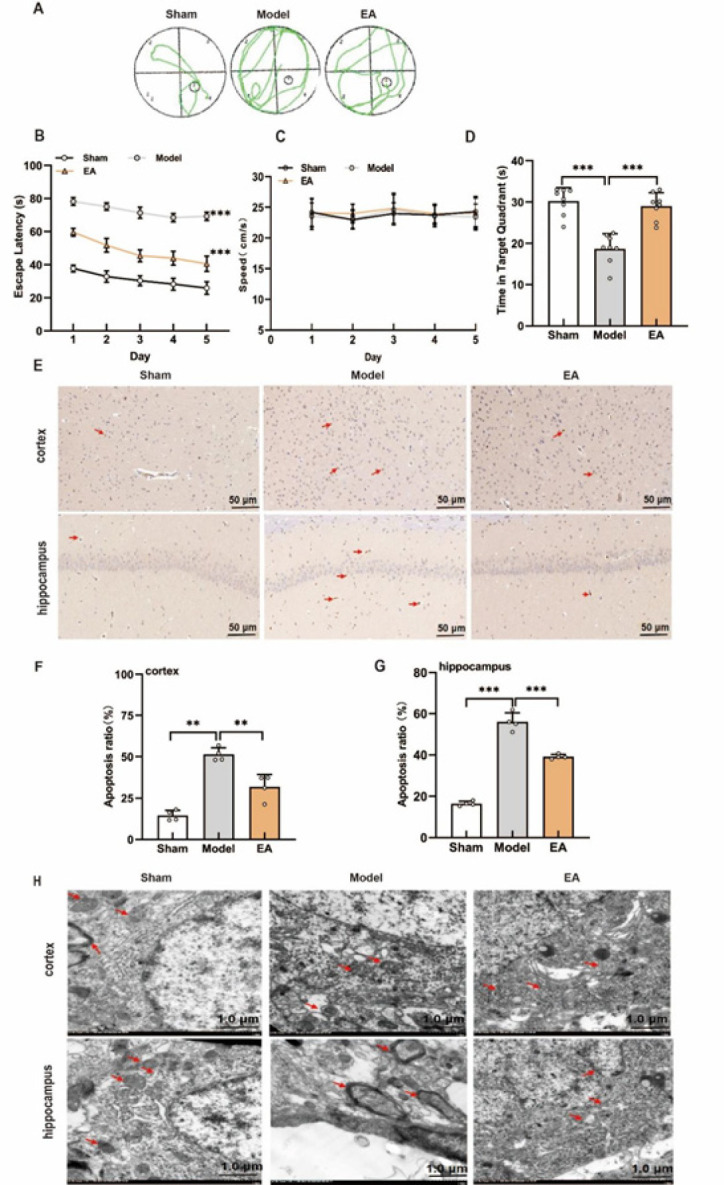
Acupoint locations and experimental design in a Sprague-Dawley rat model of vascular cognitive impairment

(A) Locations of the Baihui (GV 20) and Fengfu (GV 16) acupoints. (B) Detailed time course schema of the experiments. BCCAO: Bilateral common carotid artery occlusion; EA: Electroacupuncture; 2ME2: 2-methoxyestradiol.

**Figure 2:**
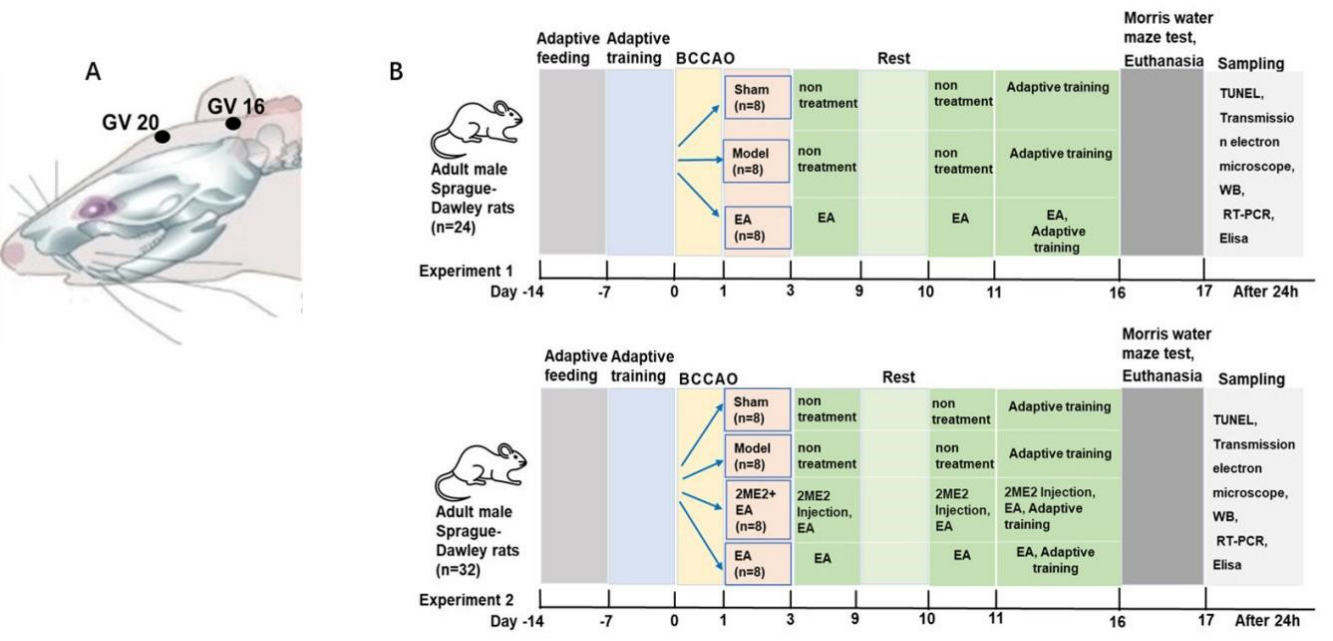
Therapeutic effect of EA on the VCI model rats

(A) Representative swimming trajectory plots for the Morris water maze test, (B) escape latency, (C) the swimming speed and (D) time spent in the target quadrant on Day 6 (n=8 rats for each group). (E–G) Representative TUNEL staining results and apoptosis ratios in the cortex and hippocampus. (H) Ultrastructures of the cortex and hippocampus (n=4 rats for each group). The values are expressed as the means ± SDs. **P<0.01 and ***P<0.001. EA: Electroacupuncture; VCI: Vascular cognitive impairment

**Figure 3:**
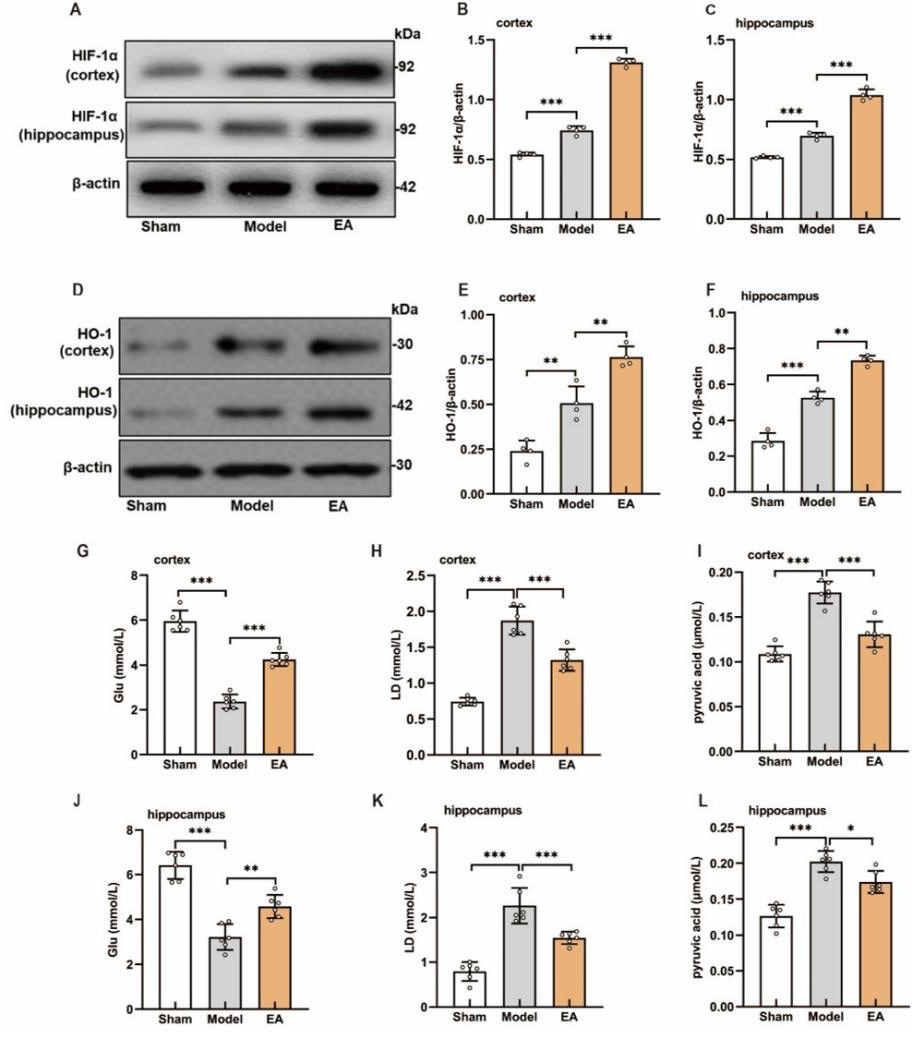
Effects of EA on hypoxia stress-related proteins and energy metabolism in the cortex and hippocampus of VCI model rats

(A, D) Representative Western blotting images. The expression levels of HIF-1α (B, C) and HO-1 (E, F) in the cortex and hippocampus (n=4 rats for each group). The concentrations of glucose (G, J), lactic acid (H, K), and pyruvic acid (I, L) in the cortex and hippocampus (n=6 rats for each group). The values are expressed as the means ± SDs. *P<0.05, **P<0.01, and ***P<0.001. EA: Electroacupuncture; VCI: Vascular cognitive impairment

**Figure 4:**
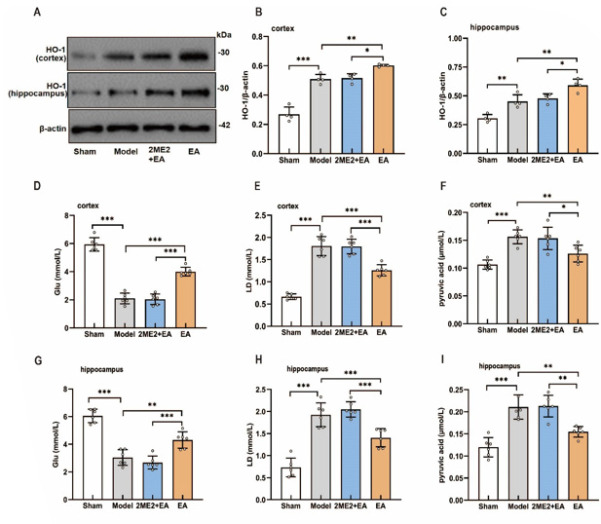
Effects of a HIF-1α inhibitor on the effects of EA treatment in VCI model rats

(A) Representative swimming trajectory plots for the Morris water maze test, (B) escape latency, (C) the swimming speed and (D) time spent in the target quadrant (n=8 rats for each group). (E) Representative TUNEL staining and apoptosis ratios in the cortex and hippocampus (F, G) (n=4 rats for each group). (H) The ultrastructures of the cortex and hippocampus. The values are expressed as the means ± SDs. ***P<0.001. EA: Electroacupuncture; VCI: Vascular cognitive impairment; HIF-1α: Hypoxia-inducible factor-1α

**Figure 5:**
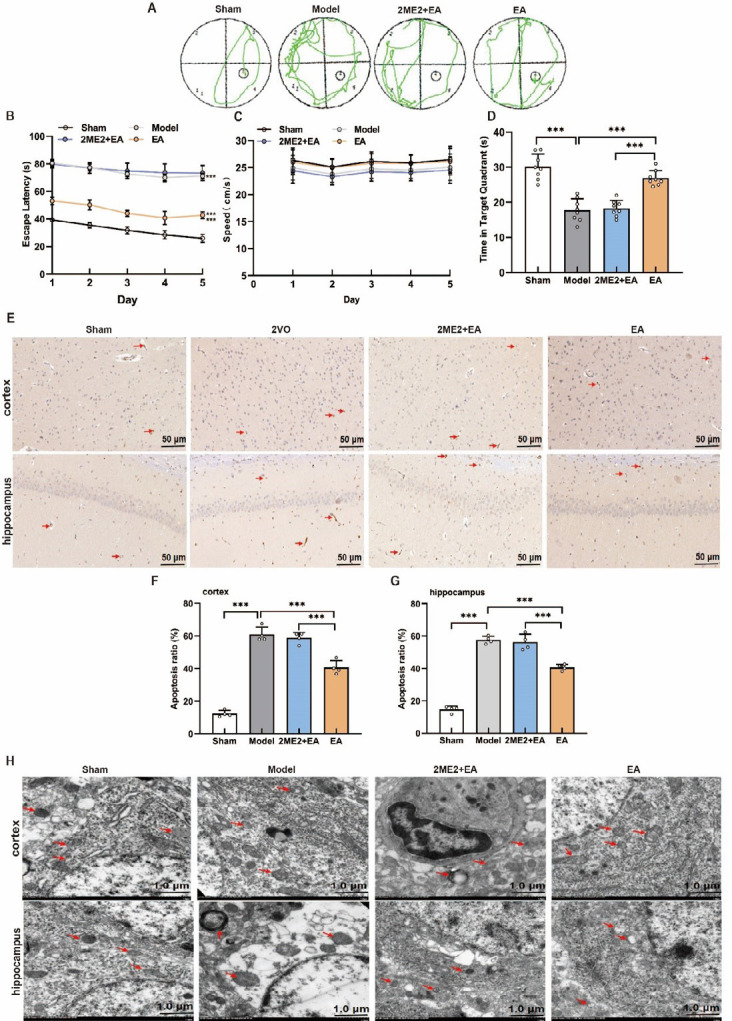
Effects of a HIF-1α inhibitor on hypoxic stress and energy metabolism in the cortex and hippocampus of VCI model rats

(A) Representative Western blotting images. (B, C) HO-1 expression in the cortex and hippocampus (n=4 rats for each group). (D, G) The levels of glucose, lactic acid (E, H) and pyruvic acid (F, I) in the cortex and hippocampus (n=6 rats for each group). The values are expressed as the means ± SDs. *P<0.05, **P<0.01 and ***P<0.001. VCI: Vascular cognitive impairment; HIF-1α: Hypoxia-inducible factor-1α

**Figure 6:**
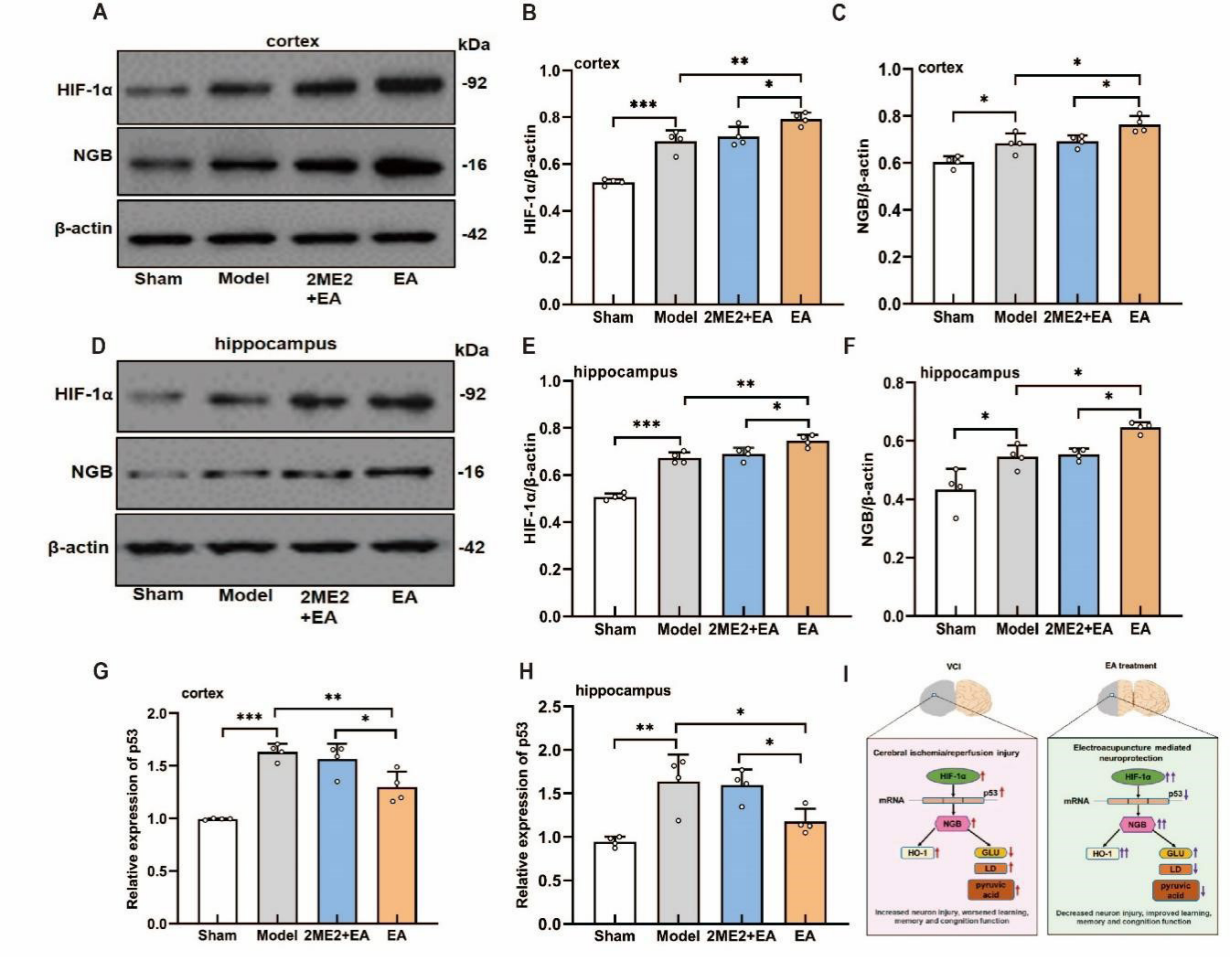
Effects of 2ME2 on HIF-1α/p53/NGB signaling in the cortex and hippocampus of VCI model rats

(A, D) Representative Western blotting images. The expression of HIF-1α (B, E), NGB (C, F) and p53 mRNA (G, H) in the cortex and hippocampus (n=4 rats for each group). (I) Schematic diagram of the regulation of HIF-1α/p53/NGB signaling by EA. The values are expressed as the means ± SDs. *P<0.05, **P<0.01, and ***P<0.001. VCI: Vascular cognitive impairment; HIF-1α: Hypoxia-inducible factor-1α; NGB: Neuroglobin; EA: Electroacupuncture

## Conclusion

In summary, the results of this study demonstrate that EA alleviates cortical and hippocampal damage induced by CCH in rats, thereby improving cognitive function. The underlying mechanism may involve activation of the HIF-1α/p53/NGB signaling pathway, which boosts the hypoxic stress response and optimizes brain energy metabolism. These findings provide experimental evidence supporting the use of acupuncture as a potential therapeutic strategy for VCI.

## Declaration

We have not utilized any AI tools or technologies in the preparation of this manuscript.
