# Supplementary figures and images for: Electroacupuncture improves hypoxic stress and energy metabolism to alleviate vascular cognitive impairment through activation of the HIF-1α/p53/NGB signaling pathway in rats
Source: Iran J Basic Med Sci. 2026;29(1):34–42. doi: 10.22038/ijbms.2025.86988.18796 (PMC12867117; doi:10.22038/ijbms.2025.86988.18796)

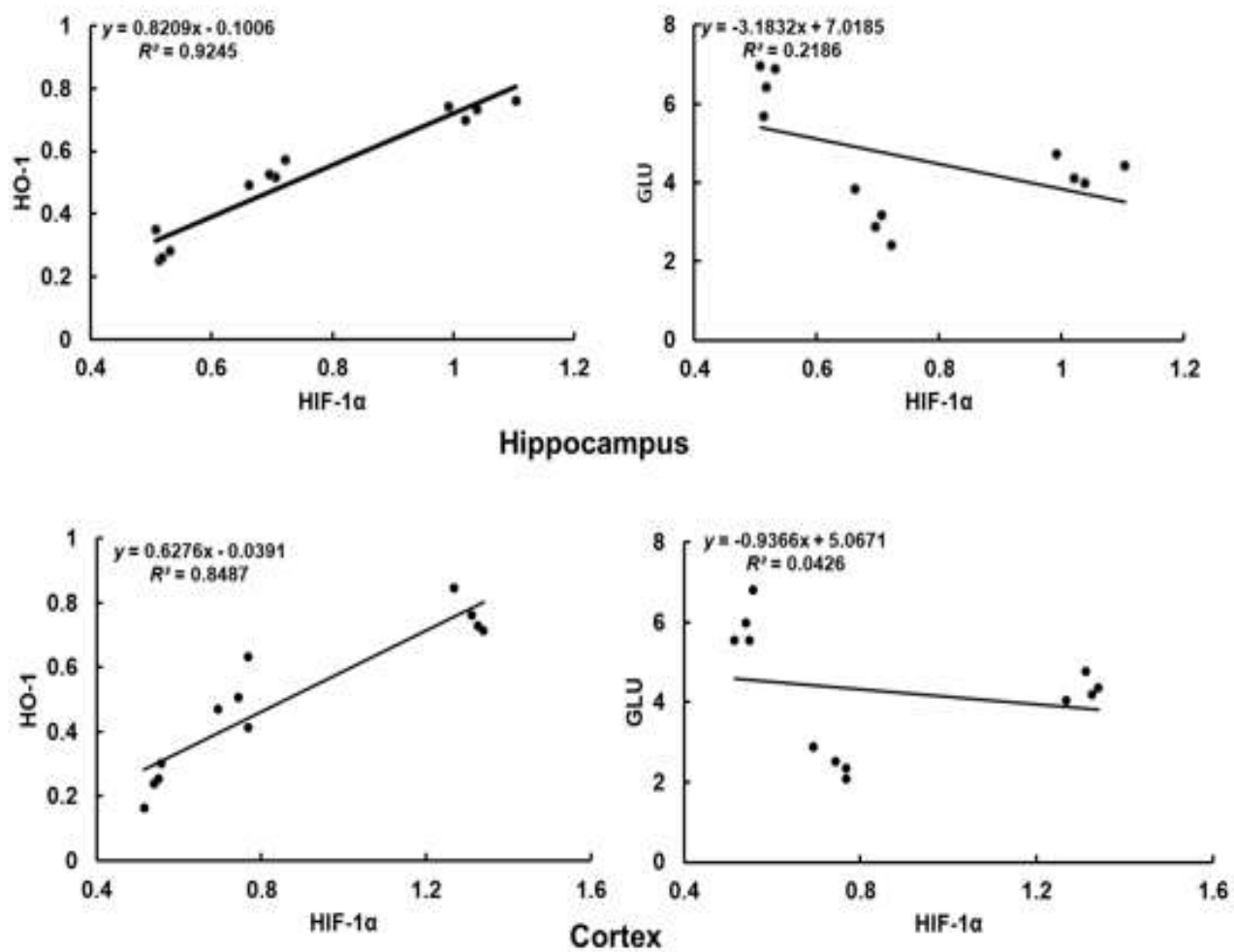

Figure S1. Correlation analysis between HIF-1α and HO-1, Glu

Supplement: Supplementary file 1 — Figure S1 [file ijbms-29-1-34-s001.pdf]
